# Investigating phenotypic relationships in persimmon accessions through integrated proteomic and metabolomic analysis of corresponding fruits

**DOI:** 10.3389/fpls.2023.1093074

**Published:** 2023-01-30

**Authors:** Sabrina De Pascale, Antonio Dario Troise, Milena Petriccione, Angelina Nunziata, Danilo Cice, Anna Magri, Anna Maria Salzano, Andrea Scaloni

**Affiliations:** ^1^ Proteomics, Metabolomics and Mass Spectrometry Laboratory, ISPAAM, National Research Council, Portici, Italy; ^2^ Consiglio per la Ricerca in Agricoltura e l’Analisi dell’Economia Agraria, Research Centre for Olive, Fruit and Citrus Crops, Caserta, Italy; ^3^ Department of Environmental Biological and Pharmaceutical Sciences and Technologies, University of Campania “Luigi Vanvitelli”, Caserta, Italy

**Keywords:** proteomics, metabolomics, biodiversity, phenotypic relationships, persimmon (*Diospyros kaki Thunb.*), fruit

## Abstract

Together with phenological and genomic approaches, gel-based and label-free proteomic as well metabolomic procedures were separately applied to plants to highlight differences between ecotypes, to estimate genetic variability within/between organism populations, or to characterize specific mutants/genetically modified lines at metabolic level. To investigate the possible use of tandem mass tag (TMT)-based quantitative proteomics in the above-mentioned contexts and based on the absence of combined proteo-metabolomic studies on *Diospyros kaki* cultivars, we here applied integrated proteomic and metabolomic approaches to fruits from Italian persimmon ecotypes with the aim to characterize plant phenotypic diversity at molecular level. We identified 2255 proteins in fruits, assigning 102 differentially represented components between cultivars, including some related to pomological, nutritional and allergenic characteristics. Thirty-three polyphenols were also identified and quantified, which belong to hydroxybenzoic acid, flavanol, hydroxycinnamic acid, flavonol, flavanone and dihydrochalcone sub-classes. Heat-map representation of quantitative proteomic and metabolomic results highlighted compound representation differences in various accessions, whose elaboration through Euclidean distance functions and other linkage methods defined dendrograms establishing phenotypic relationships between cultivars. Principal component analysis of proteomic and metabolomic data provided clear information on phenotypic differences/similarities between persimmon accessions. Coherent cultivar association results were observed between proteomic and metabolomic data, emphasizing the utility of integrating combined *omic* approaches to identify and validate phenotypic relationships between ecotypes, and to estimate corresponding variability and distance. Accordingly, this study describes an original, combined approach to outline phenotypic signatures in persimmon cultivars, which may be used for a further characterization of other ecotypes of the same species and an improved description of nutritional characteristics of corresponding fruits.

## 1 Introduction

Persimmon (*Diospyros kaki* Thunb.) (2n=6x=90) is a perennial woody tree and the most economically important cultivated species of the genus *Diospyros*, whose palatable fruits are appreciated worldwide (Yonemori et al., 2000). This plant is believed to have originated about 25 million years ago in China (Wu, 2005), where its cultivation has found a wide development overtime (Wang et al., 2013). Persimmon was then introduced in Japan and Korea (15^th^ century), and more recently in Europe, Australia, Brazil and United States (Yonemori et al., 2000). This plant grows well in southern climates, but varieties also exist that tolerate cold conditions (about -15°C) for short periods. In general, optimal plant growth occurs in fertile and well-drained soils having a pH value between 6.5-7.5, and within the temperature range 13-19°C. Italy produces about 55.000 tons of persimmon fruits each year, as deriving from national cultivars, including the most common variety Kaki tipo (Zhang and Luo, 2022; Insero et al., 2002a) and the local ecotypes Vaniglia, Lampadina and Ciccolatino, the latter ones being also used as pollinators of the former accession (Insero et al., 2002b). Recently, local cultivars have been the object of dedicated regional interventions to sustain agrobiodiversity and long-term strategies protecting uncommon accessions.

Most persimmon fruits accumulate large amounts of proanthocyanidins (PAs), which are synthesized *via* the shikimate and flavonoid pathways (Xie and Dixon, 2005; Lepiniec et al., 2006), and cause a strong astringency sensation in fresh crops, even at commercial maturity (Wu et al., 2022). In this case, fruits arouse consumer interest only after artificial treatments to remove astringency, which are generally based on the application of acetaldehyde, ethanol, CO_2_, or warm water soaking (Wu et al., 2022). Spontaneous plant mutant phenotypes also exist whose fruits drop their astringency and become palatable directly on the tree during ripening (Yonemori et al., 2000).

*D. kaki* is a hexaploid plant that was proposed to originate through polyploidization of diploid ancestors (Kanzaki, 2016). Less diffused diploid *D. lotus* and *D. oleifera* species (2n=2x=60) are also cultivated for fruit production, and for root-stock and oil production applications, respectively (Guo and Luo, 2011). To provide significant insights into formation/removal of fruit astringency as well as the genetic basis of hexaploid *D. kaki* evolution, above-mentioned diploid species were subjected to genome sequencing. By integrating data from multiple sequencing approaches, the genome of *D. oleifera* was assembled, assigning genes to 15 pseudochromosomes and identifying this species as one of *D. kaki* ancestors (Zhu et al., 2019; Suo et al., 2020). This genome also provided information on the genetic basis for astringency development and removal. PAs biosynthesis genes were clustered in specific regions of chromosome 1; deastringency genes were also identified, whose promoters showed low O_2_-responsive motifs. Similar findings and novel information on the evolution of the plant lineage-specific sex determination system were achieved after the assembly of the genome of *D. lotus* (Akagi et al., 2020). Concomitant transcriptomic investigations also identified elements of genetic divergence, and domestication genes in various *Diospyros* species (Guan et al., 2019).

Several transcriptomic studies were accomplished on *D. kaki* to identify regulatory networks and protein regulators associated with tannin metabolism in cultivars showing variable PAs accumulation patterns (Jung et al., 2017; Nishiyama et al., 2018; Zheng et al., 2021), or to reveal deregulated metabolic pathways in fruits at different developmental (Zhang and Luo, 2022) and post-harvest (Kou et al., 2021a) stages, or following treatment with ethanol (Luo et al., 2014), warm water (Chen et al., 2017) and CO_2_ (Kou et al., 2021b). Various key genes affecting PAs formation and removal were identified. On the other hand, different metabolomic approaches based on NMR or liquid chromatography-tandem mass spectrometry (LC-ESI-MS/MS) were used to investigate metabolite concentration variations in various non-Italian *D. kaki* cultivars (Maulidiani et al., 2018; Santos et al., 2018; Ryu et al., 2019; Esteban-Munoz et al., 2020), with the aim to identify peculiar composition differences among them, and detail at molecular level intraspecies biodiversity.

Together with phenological and genomic approaches, gel-based proteomics was successfully utilized in the past to describe difference between plant ecotypes, estimate genetic variability within/between organism populations, establish genetic distances for phylogenetic studies, and characterize specific mutants/genetically modified lines grown under identical experimental conditions by using corresponding two-dimensional (2D) electrophoretic maps (Thiellement et al., 1999). Dedicated studies were accomplished on proteins extracted from roots and seeds of *Arabidopsis thaliana* (Chevalier et al., 2004; Ruebelt et al., 2006), tubers of potato (Lehesranta et al., 2005), seeds of lentil (Ialicicco et al., 2012), seeds of soybean (Gomes et al., 2014; Min et al., 2015), grains of bean (Rossi et al., 2017) and seeds of wheat (Croes et al., 2009; Gao et al., 2009) accessions. Quali-quantitative data on protein spot volumes from 2D electrophoretic maps allowed computing cluster analysis on the correlation matrix for different ecotypes as well as calculating corresponding principal component analysis (PCA) plot diagrams. In some cases, unrooted phenetic trees were built from the distance matrix calculated according to the Jaccard index on all spots from the analyzed cultivars using neighbor-joining algorithms. Recently, label-free shotgun proteomics has been used to investigate quali-quantitative differences between root, seed, or fruit tissues from different pea (Meisrimler et al., 2017), grape (Carpentieri et al., 2019), quinoa (Galindo-Lujan et al., 2021), and narrow-leafed lupin (Tahmasian et al., 2022) accessions, respectively. Statistical approaches generated peculiar heat-maps reporting quantitative protein profiles of cultivars, whose dendrogram from unsupervised hierarchical cluster analysis described the similarity of accessions, whereas PCA score biplot diagrams of protein percentage data among them illustrated phenotype relationships.

Notwithstanding its precise and accurate characteristics (Li et al., 2012; Megger et al., 2014) but probably as result of the possible limited enhancement of its sensitivity due to the need of peptide derivatization (Bachor et al., 2019), no tandem mass tag (TMT)-based quantitative proteomic procedures have been applied yet to evaluate genetic differences, variability, and distance in plant cultivars. To establish whether this technology is suitable to above-mentioned studies and due to the absence of integrated proteo-metabolomic information on *D. kaki* allowing a distinction of corresponding ecotypes at molecular level, we have here investigated the biodiversity of four persimmon Italian accessions through combined TMT-based proteomics and metabolomics applied to corresponding fruits. Bioinformatic analysis of resulting data generated novel information in the above-mentioned context and defined phenotypic relationships for these cultivars. Coherent cultivar association results were observed between proteomic and metabolomic data, which emphasized the value of integrating results from different *omic* approaches to validate information at molecular level.

## 2 Materials and methods

### 2.1 Chemicals

Methanol, acetonitrile and water were of mass spectrometry grade and were obtained from Merck Sigma-Aldrich (Darmstadt, Germany). Trolox reagent, potassium persulfate, sodium chloride, formic acid, sodium dihydrogen phosphate dihydrate, di-sodium hydrogen phosphate dihydrate and the Folin-Ciocalteu′s reagent was purchased from Merck Sigma-Aldrich. All the other chemicals were of analytical grade and were obtained from Merck Sigma-Aldrich unless otherwise indicated.

### 2.2 Fruit material

Persimmon fruits (n=80) having similar distribution over the tree, similar size and identical maturation degree (about 145 days post-anthesis) were randomly harvested from five trees of each Kaki tipo, Vaniglia, Lampadina and Cioccolatino cultivars grown closely in the same field located in Santa Agata dei Goti (Caserta) (Italy; 41°05’N, 14°27°E), under standard practice in the management of the orchard, which experienced a temperature ranging from -5.0 to 35.2°C, a humidity ranging from 97% to 25%, and a dough soil; above-mentioned cultivars are all pollination variant and non-astringent ecotypes. Only pollinated fruits without astringency at harvest were collected and selected in the laboratory for the absence of qualitative/mechanical defects and were subjected to further analyses. Above-reported persimmon cultivars were described using the 50 descriptors defined by the International Union for the Protection of New Varieties (TG_92_4; UPOV, 2004) (**Supplementary Table S1**).

### 2.3 Physicochemical characterization of fruits

Fruit firmness was determined at two equatorial points of 15 fruits with a digital penetrometer (Model TR-Turoni, Italy) equipped with an 8 mm-flat tip; results are expressed as Newton (N) values. Flesh juice was obtained from 10 fruits with an electric juice extractor and filtered. Total soluble solid content (SCC) was measured using a digital refractometer (Sinergica Soluzioni, DBR35, Pescara, Italy). Total acid content (TA) was evaluated by titration with 0.1 M NaOH; results are expressed as malic acid (g)/L. The skin color was assessed using a Minolta colorimeter (Model CR5, Minolta Camera Co., Japan) based on CIE L*, a*, b* mode at two equatorial points of ten fruits; L* indicates the lightness or darkness; a* is green or red color of the samples; and b* is blue or yellow color of them. The Folin–Ciocalteu method was used to evaluate the total phenolic compound (POL) content in samples (Goffi et al., 2020). POL results were expressed as µmol of gallic acid equivalents (GAE) per g of fresh weight (FW). Antioxidant activity (AOX) was detected using the 2,2-diphenyl-2-picryl-hydrazil (DPPH) method, with some modifications (Goffi et al., 2020). In this case, the reaction mixture contained 62.5 µM DPPH and 100 µL of fruit extract. The results were expressed as µmol Trolox equivalent (TE) per g of FW. Total protein (TP) content in fruit was determined by Kjeldahl method according to ISO 1871:2009 guidelines (ISO 1871:2009, 2009). After sample mineralization with a Foss Tecator™ 2508 device (Fisher Scientific, USA), total nitrogen content was measured with a Foss Kjeltec™ 8200 instrument (Fisher Scientific, USA).

Physicochemical data are reported as mean values ± standard deviation and were analyzed by one-way ANOVA. Mean values were compared by Tukey’s test with a level of significance α = 0.05 using SPSS software package, v. 20.0 (SPSS, Chicago, IL, USA).

### 2.4 Protein extraction

Fruit samples were freeze-dried and crushed using a mortar containing liquid N_2_ to obtain a fine powder. Proteins were extracted in parallel through a modified version of the phenol extraction method (Saravanan and Rose, 2004). Thus, 1 g of fine powder of each sample added with 0.01 g of polyvinylpyrrolidone was resuspended in a buffer consisting in 0.7 M sucrose, 0.1 M KCl, 0.5 M Tris-HCl (pH 8.8), 50 mM EDTA, 40 mM DL-dithiothreitol, 1 mM phenylmethylsulfonyl fluoride, plus 30 µL of protease inhibitors cocktail for plant tissues (Sigma-Aldrich, USA). Three independent biological replicates were analyzed in comparison for each cultivar. The mixtures were homogenized with T10 basic Ultra-Turrax™ (IKA, Staufen, Germany) with 5 cycles (20,000 rpm) of 10 sec, and 20-sec pauses on ice. Afterward, an equal volume of saturated phenol in 0.5 M Tris-HCl, pH 8.1 (Sigma-Aldrich) was added, and the solution was incubated on a shaker for 20 min, at 4°C. The samples were centrifuged at 10,000 *g* for 10 min, at 4°C. The phenolic phase was recovered carefully to avoid contact with the interphase and poured into a new tube. This phenol phase was then back-extracted with 3 mL of resuspension buffer. All samples were shaken for 3 min and then vortexed. The phenolic phase was further recovered by centrifugation at 10000 g for 10 min, at 4°C. Proteins were precipitated by adding six vol of ice-cold 0.1 M ammonium acetate in methanol, overnight, at -20°C. Protein pellets were then washed three times with ice-cold 0.1 M ammonium acetate in methanol, and three times with ice-cold acetone. Protein pellets were air-dried and then solubilized in 600 μL of 8 M urea, 50 mM triethylammonium bicarbonate (TEAB), pH 8.5. Samples were vortexed, sonicated in ultrasonic bath for 5 min, and left on a shaker at 22°C, overnight. Samples were centrifuged at 12,000 rpm for 30 min, at 22°C, and protein concentration was determined on the supernatant using the Pierce BCA Protein assay kit™ (Thermo Scientific, Rockford, IL, USA), according to manufacturer’s instructions. The purity and overall quality of protein extracts were evaluated with Laemmli buffer-including SDS-PAGE (Laemmli, 1970). Electrophoresis was conducted using a SE600 Chroma™ device (Hoefer, Holliston, USA). The gel was then stained with Coomassie Brilliant Blue G-250 (Bio-Rad, Hercules, CA).

### 2.5 Protein identification and relative quantitation

Quantitative proteomic analysis of persimmon fruits was performed according to the tandem mass tag (TMT) approach, as already reported for other plant tissues (Lombardi et al., 2020; Samperna et al., 2021). In detail, an aliquot of each extracted protein sample (100 μg) was adjusted to 100 μL final volume with 100 mM TEAB, and then reduced with 5 μl of 200 mM tris(2-carboxyethylphosphine), for 60 min, at 55°C. Protein samples were then alkylated by adding 5 μL of 375 mM iodoacetamide in the dark, for 30 min, at 25°C. To remove chemicals, alkylated proteins were precipitated by addition of 6 vol of ice-cold acetone, overnight, at -20°C. Proteins were pelleted by centrifugation at 8000 g, for 10 min, at 4°C, and then air-dried. Each sample was digested with freshly prepared trypsin (enzyme to protein ratio 1:50 w/w) in 100 mM TEAB, at 37°C, overnight. Resulting peptides from each protein sample were labelled with the TMT6plex Label Reagent Set (Thermo-Fisher Scientific, USA) following the manufacturer’s instructions, according to the scheme: Cioccolatino TMT-126, Vaniglia TMT-127, Lampadina TMT-128N, Kaki tipo TMT-129. After 1 h, 8 μL of 5% w/v hydroxylamine was added in each tube and mixed for 15 min to quench the labeling reaction. For a set of comparative experiments, tagged peptide mixtures were mixed in equal-molar ratios (1:1:1:1) and vacuum-dried by using a centrifugal evaporator (SpeedVac, Thermo Fisher Scientific, Bremen, Germany). To remove unbound TMT reagents and reduce sample complexity, pooled TMT-labelled peptide mixtures were then suspended in 0.1% trifluoroacetic acid and fractionated by using the Pierce™ High pH Reversed-Phase Peptide fractionation kit (Thermo-Fisher Scientific) according to manufacturer’s instructions. After fractionation, eight fractions of TMT-labelled peptides were collected, vacuum-dried and finally reconstituted in 0.1% formic acid for subsequent mass spectrometric analysis. TMT-labelled peptide fractions (eight in total number as deriving from three independent biological replicates of four persimmon cultivars) were analyzed on a nanoLC-ESI-Q-Orbitrap-MS/MS platform consisting of an UltiMate 3000 HPLC RSLCnano system (Dionex, USA) coupled to a Q-ExactivePlus mass spectrometer through a Nanoflex ion source (Thermo Fisher Scientific). Peptides were loaded on an Acclaim PepMapTM RSLC C18 column (150 mm ×75 μm ID, 2 μm particles, 100 Å pore size) (Thermo-Fisher Scientific), and eluted with a gradient of solvent B (water/acetonitrile/formic acid 19.92/80/0.08 v/v/v) in solvent A (water/formic acid 99.9/0.1 v/v), at a flow rate of 300 nL/min. After an equilibration step of 20 min at 5% of solvent B, the gradient increased to 60% over 125 min, raised to 95% over 1 min, remained at 95% for 8 min, and finally returned to 5% in 1 min. The mass spectrometer operated in data-dependent mode, using a full scan (*m/z* range 375–1500, nominal resolution of 70,000), followed by MS/MS scans of the 10 most abundant ions. MS/MS spectra were acquired in a scan *m/z* range 110–2000, using a normalized collision energy of 32%, an automatic gain control target of 100,000, a maximum ion target of 120 ms, and a resolution of 17,500. A dynamic exclusion value of 30 s was also used.

### 2.6 Bioinformatic analysis of proteomic data

Raw data files were analyzed for protein identification and relative quantification by using Proteome Discoverer vs 2.4 (PD2.4) software (Thermo Scientific), enabling the database search by Mascot algorithm v. 2.6.1 (Matrix Science, UK) using the *D*. *lotus* protein database (51693 protein sequences) deriving from the corresponding sequenced genome (Akagi et al., 2020), which was added with most common protein contaminants. A similar protein database was not available for *D. oleifera* (Chen et al., 2017; Zhu et al., 2019; Suo et al., 2020). Database searching was performed using following criteria: carbamidomethylation of Cys and TMT6-plex modification of lysine and peptide N-terminal as fixed modifications; oxidation of Met, deamidation of Asn and Gln, pyroglutamate formation of Gln/Glu as variable modifications. Peptide mass tolerance was set to ± 10 ppm and fragment mass tolerance to ± 0.05 Da. Proteolytic enzyme and maximum number of missed cleavages were set to trypsin and 2, respectively. Protein candidates were considered confidently identified based on at least two sequenced peptides and an individual Mascot Score greater or equal to 30. Results were filtered to 1% false discovery rate. Protein abundance values were obtained from TMT reporter ion intensities in the MS/MS spectra, which were subjected to PD2.4 software elaboration using one-way ANOVA. Results were finally filtered to retain only proteins that were significantly changed in at least one by one comparison between cultivars, considering an abundance ratio *p*-value ≤ 0.01 and a fold change of abundance > ± 1.2. Proteomic data were deposited to the ProteomeXchange Consortium *via* the PRIDE partner repository (Perez-Riverol et al., 2022) with the dataset identifier PXD037485.

Visualization of quantitative patterns across the differentially represented proteins (DRPs) and cultivars was obtained by heat-map elaboration of data, which was performed with PD2.4 software using Euclidean distance and average linkage method. In parallel, PCA analysis of proteomic results was performed with PD2.4 software and included identified DRPs as variables and persimmon cultivars as factors, which were correlated in a two-dimensional spatial distribution.

Functional analysis of the DRPs was performed as previously reported (Salzano et al., 2019). A preliminary functional classification was obtained following data analysis with Mercator4 v5.0 software (Lohse et al., 2014), which was further integrated with information from recent literature data.

### 2.7 Screening for potential allergenic sequences

Putative identification of persimmon allergens was obtained carrying out a Basic Local Alignment Search Tool (BLAST) analysis against a database of 2463 known allergens obtained from the COMprehensive Protein Allergen REsource database (van Ree et al., 2021), using command-line applications developed at the National Center for Biotechnology Information. The results of BLAST analysis were filtered to retain only homologous proteins showing at least 80% sequence identity.

### 2.8 Metabolite extraction

Polyphenols were extracted from fruits through a dedicated procedure already set up for persimmon (Ancillotti et al., 2018). Briefly, freeze-dried persimmon samples were pulverized by using a knife-mill Grindomix GM200 (Retsch, Haan, Germany), and 0.2 g were mixed with 1 mL of methanol/water 70:30 v/v. Suspensions were vortexed (5 min, 1000 rpm) and sonicated in an ice bath for 15 min. Triplicate samples of each cultivar were independently centrifuged (2,600 g) and hydroalcoholic supernatants were collected for reversed phase solid phase extraction. Polymeric C18 cartridges (Strata-X 30 mg, Phenomenex, Torrance, CA) were activated with 3 mL of methanol and 3 mL of water; upon ten times dilution in 0.1% formic acid in water, samples were spiked with 10 μg/mL of 4-butyl-hydroxybenzoate and then loaded on the cartridges. The latter ones were washed with 2 mL of 0.1% formic acid and then eluted with 1 mL of methanol to collect the corresponding polyphenolic fraction. Samples were dried in a centrifugal evaporator and then dissolved in water/methanol 85:15 v/v for further LC-ESI-MS/MS analysis.

### 2.9 Metabolomic analysis

Mass spectrometry data were acquired by using a LTQ Orbitrap XL interfaced to an Ultimate 3000 RS (Thermo Fisher Scientific). Polyphenol separation was achieved through a thermostated (35°C) core-shell reversed phase column with a positive charge surface (Kinetex PS C18, 100 x 2.1 mm, 2.6 µm, Phenomenex). Mobile phases consisted in 0.1% formic acid in water (solvent A) and 0.1% formic acid in methanol (solvent B). Samples (5 µL) were injected in full loop mode and analytes were resolved through the following gradient of solvent B (minutes/%B): (0/10), (2/10), (12.5/55), (14/55), (17/95), (19/95), at a flow rate of 0.2 mL/min, with an equilibration stage of 6.5 min at 10% of mobile phase B. Analytes were screened in negative ion top four untargeted data dependent scanning mode; ESI interface spray voltage and capillary voltage were -4.2 kV and -70.0 V, respectively. Capillary temperature was 275°C; sheath gas and auxiliary gas flow values were 25 and 3 arbitrary units, respectively. Profile data type were acquired in full scan Fourier transformed mass spectrometry (FTMS) mode in the mass scanning *m/z* range 70–1200. For data dependent scanning, MS/MS normalized collision energy was 25, activation Q was 0.25, activation time was 25 ms, with a 1 *m/z* isolation window, while a reject mass list was generated by injecting blank samples.

### 2.10 Bioinformatic analysis of metabolomic data

Identification of polyphenols was achieved through the matching of the analyte retention time, chemical formula, exact mass and MS/MS spectra with publicly available database mzCloud (www.mzcloud.org), Phenol-explorer (www.phenol-explorer.eu), FooDB (www.foodb.ca), and PhytoHub (www.phytohub.eu). MS/MS spectra were manually annotated and curated for the best fragmentation pattern to identify correspondences with database spectra. Blank samples were used to exclude noise and impurities, and to subtract background signals; quality controls of pooled samples were used to correct signal intensities in FTMS mode for a final import in an *in-house* mass list generated in Xcalibur 2.1 (Thermo Fisher Scientific).

Area counts of target analytes were loaded in XLStat (v. 5.03, Addinsoft, NY, USA) for multivariate data analysis. PCA transformed normalized area counts of polyphenols into a spatial distribution set of independent linear combinations of the polyphenol classes as principal components and persimmon cultivars as factors; polyphenol classes and cultivars were related through Pearson correlation. In parallel, red to blue through white heat-map was optimized through centering and reduction procedures to magnify association between cultivars (dendrograms on x axis) and analytes (dendrograms on y axis) following Euclidean distance and the Ward linkage method.

## 3 Results and discussion

### 3.1 Physicochemical characteristics of fruit of persimmon ecotypes

**Figure 1:**
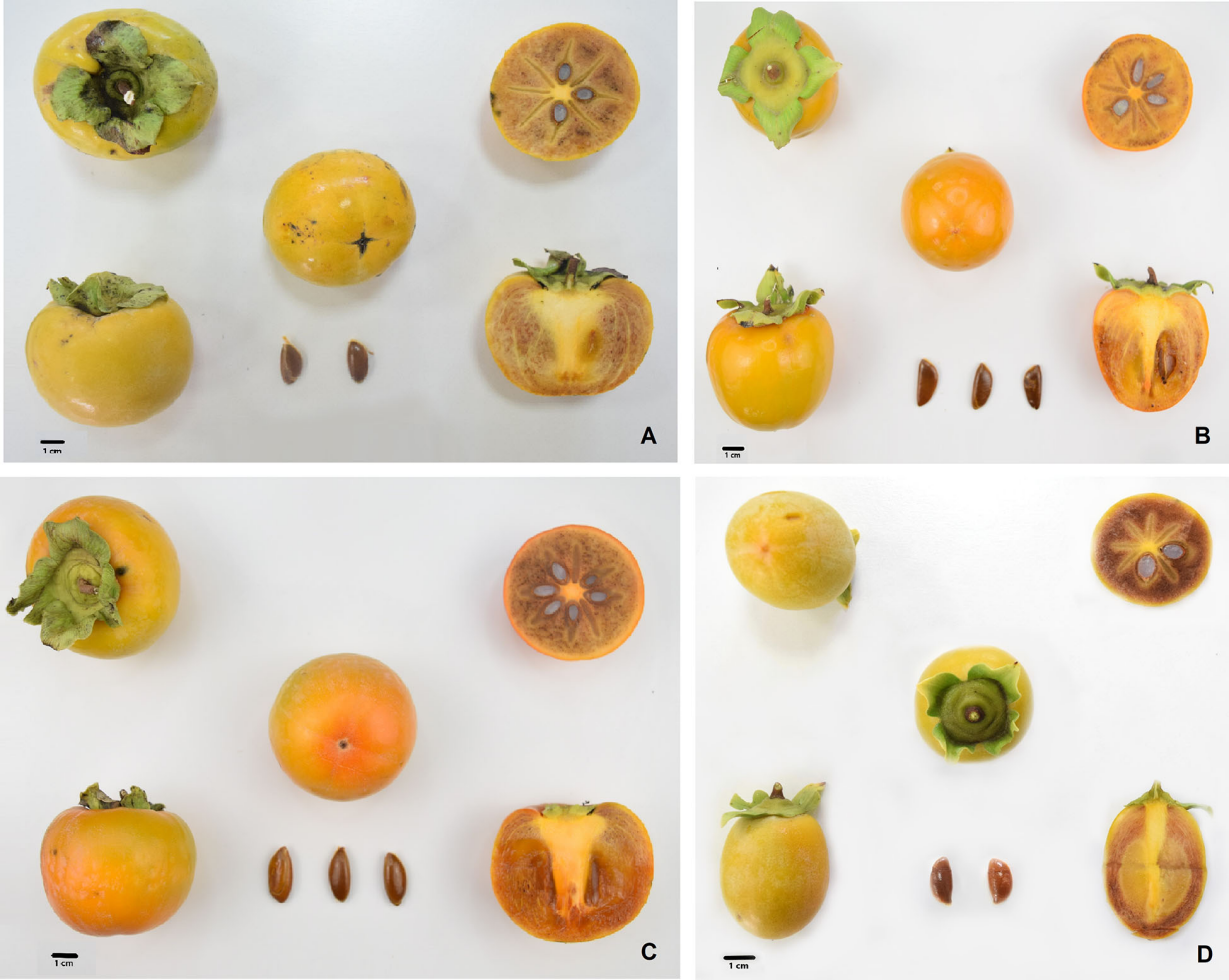
Morphological characteristics of persimmon fruits of Kaki tipo, Cioccolatino, Vaniglia and Lampadina cultivars. **(A)** Kaki tipo; **(B)** Cioccolatino; **(C)** Vaniglia; **(D)** Lampadina. Bars correspond to a 1 cm-length.

The phenological and agronomic traits of Kaki tipo, Lampadina, Vaniglia and Cioccolatino plant ecotypes were originally characterized and described according to International Union for the Protection of New Varieties of Plants (UPOV) descriptors (**Supplementary Table S1**). Pollinated persimmon fruits having a very similar distribution over the tree and an identical maturation degree were harvested from plant cultivars and were further characterized with methods officially recognized to monitor the corresponding ripening. Notwithstanding minor differences between cultivars, measured parameters (**Table 1**) were in general good agreement with preliminary information already described for some of these ecotypes (Giordani, 2002; Insero et al., 2002a). Regarding physicochemical traits, the mean weight of fruit ranged from a minimum value of 95.0 ± 12.4 g for Cioccolatino ecotype to a maximum value of 174.4 ± 23.3 g for Kaki tipo one (**Table 1**). General shape in lateral view of fruits was circular for Kaki tipo and Vaniglia cultivars, elliptic for Lampadina counterpart and very broad ovate in Cioccolatino one (**Figure 1**). Total soluble solids estimate the level of dissolved sugars (SSC), which ranged from 15.2 ± 0.5 to 17.5 ± 0.6 Brix in these persimmon fruits (**Table 1**). TA showed the lowest value in Cioccolatino ecotype, while no significant differences occurred among the other cultivars. The L* coordinate measures the fruit luminosity; not significantly different of *L values were observed among different cultivars (**Table 1**). Conversely, Vaniglia and Cioccolatino ecotypes showed the highest values of a* coordinate (**Table 1**), indicating a more pronounced orange skin color compared to Lampadina and Kaki tipo counterparts, having a yellow-orange one (**Figure 1**).

**Table 1:** Pomological and fruit qualitative traits of Kaki tipo, Cioccolatino, Vaniglia and Lampadina cultivars.

Accessions	Fruit Weight (g)	Firmness (N)	L*	a*	b*	SSC (°Brix)	TA (mg malic acid/L)	pH	POL (mg GAE/ 100g FW)	AOX (µmol Trolox/ g FW)	TP (g/100 g FW)
**Lampadina**	130.1 ± 10.1^b^	7.9 ± 0.9^a^	75.7 ± 1.1^bc^	6.0 ± 1.7^a^	67.5 ± 2.3^ab^	17.0 ± 0.6^b^	2.0 ± 0.1^b^	6.15 ± 0.01^a^	65.33 ± 3.42^b^	0.61 ± 0.03^a^	0.48 ± 0.01^b^
**Vaniglia**	106.9 ± 16.5^ab^	7.8 ± 0.7^a^	71.6 ± 2.1^a^	32.6 ± 3.8^c^	60.9 ± 7.8^a^	16.6 ± 0.5^ab^	2.0 ± 0.2^b^	6.14 ± 0.01^a^	56.91 ± 2.40^a^	1.07 ± 0.02^c^	0.35 ± 0.01^a^
**Cioccolatino**	95.1 ± 12.4^a^	8.2 ± 0.8^a^	74.3 ± 1.4^b^	24.9 ± 2.7^b^	71.5 ± 3.3^b^	17.5 ± 0.6^b^	1.3 ± 0.1^a^	5.99 ± 0.02^a^	66.61 ± 0.61^b^	0.78 ± 0.07^b^	0.58 ± 0.02^c^
**Kaki Tipo**	174.4 ± 23.3^c^	8.3 ± 0.8^a^	77.6 ± 1.2^c^	7.11 ± 1.9^a^	70.4 ± 5.2^b^	15.2 ± 0.5^a^	2.1 ± 0.1^b^	5.97 ± 0.12^a^	71.06 ± 7.50^b^	5.00 ± 0.14^d^	0.55 ± 0.02^c^

Reported are data on fruit fresh weight (FW), firmness, colorimetric coordinates (L*, a* and b*), soluble sugar content (SSC), total acid content (TA), pH, total polyphenol content (POL), antioxidant activity (AOX), and total protein content (TP). GAE, gallic acid equivalents; FW, fresh weight. Data are reported as mean values ± standard deviation. Differences among cultivars within each column were analyzed by ANOVA with a confidence interval of 95%. Different letters indicate a significant difference (Tukey test, P < 0.05).

Many persimmon genotypes have been studied for their polyphenol content and antioxidant activity (Ercisli et al., 2008; Li et al., 2011). Polyphenol content in the four persimmon genotypes was comparable to data already reported for non-astringent cultivars (Li et al., 2011) (**Table 1**). The persimmon ecotype with the greatest content of polyphenols, namely Kaki tipo, also showed the highest value of antioxidant activity (**Table 1**), suggesting a good correlation with these parameters.

### 3.2 Proteomic analysis

To further investigate the trend of protein representation among the different cultivars, each persimmon ecotype lot (10 fruits) was in parallel subjected to an extraction protocol for subsequent proteomic analysis. We used about 200 g of mixed and crashed fruits for each cultivar pool; three biological replicates of each ecotype were analyzed. Fruits were treated in parallel as described in the experimental section and then extracted with trichloroacetic acid/acetone or phenol. Although with limited differences, the latter procedure provided optimal results in terms of total protein recovery and protein banding pattern representation, as evidenced by dedicated Bradford assays and SDS-PAGE analysis (data not shown); thus, it was used to prepare protein extracts for further proteomic analysis. Data on total proteins recorded with the colorimetric assay for the different cultivars followed the concentration trend values reported in **Table 1**. Equal amounts of fruit proteins were subjected to proteomic analysis, as reported in the experimental section. Resulting peptide mixtures were labeled with TMT reagents, which provided multiplexing capabilities for relative analyte quantitation (Lombardi et al., 2020; Samperna et al., 2021), combined and analyzed with nano-liquid chromatography-electrospray-quadrupole-Orbitrap-tandem mass spectrometry (nLC-ESI-Q-Orbitrap-MS/MS). Due to the remarkable speed of the quadrupole analyzer coupled to the high resolution Orbitrap device, we collected a very large data set for simultaneous protein identification and quantification. Depending on the absence of information on *D. kaki*, database searching of mass spectrometric data was performed against a non-redundant protein sequence FASTA file available for *D. lotus*, as deriving from the corresponding genome (Akagi et al., 2020).

**Figure 2:**
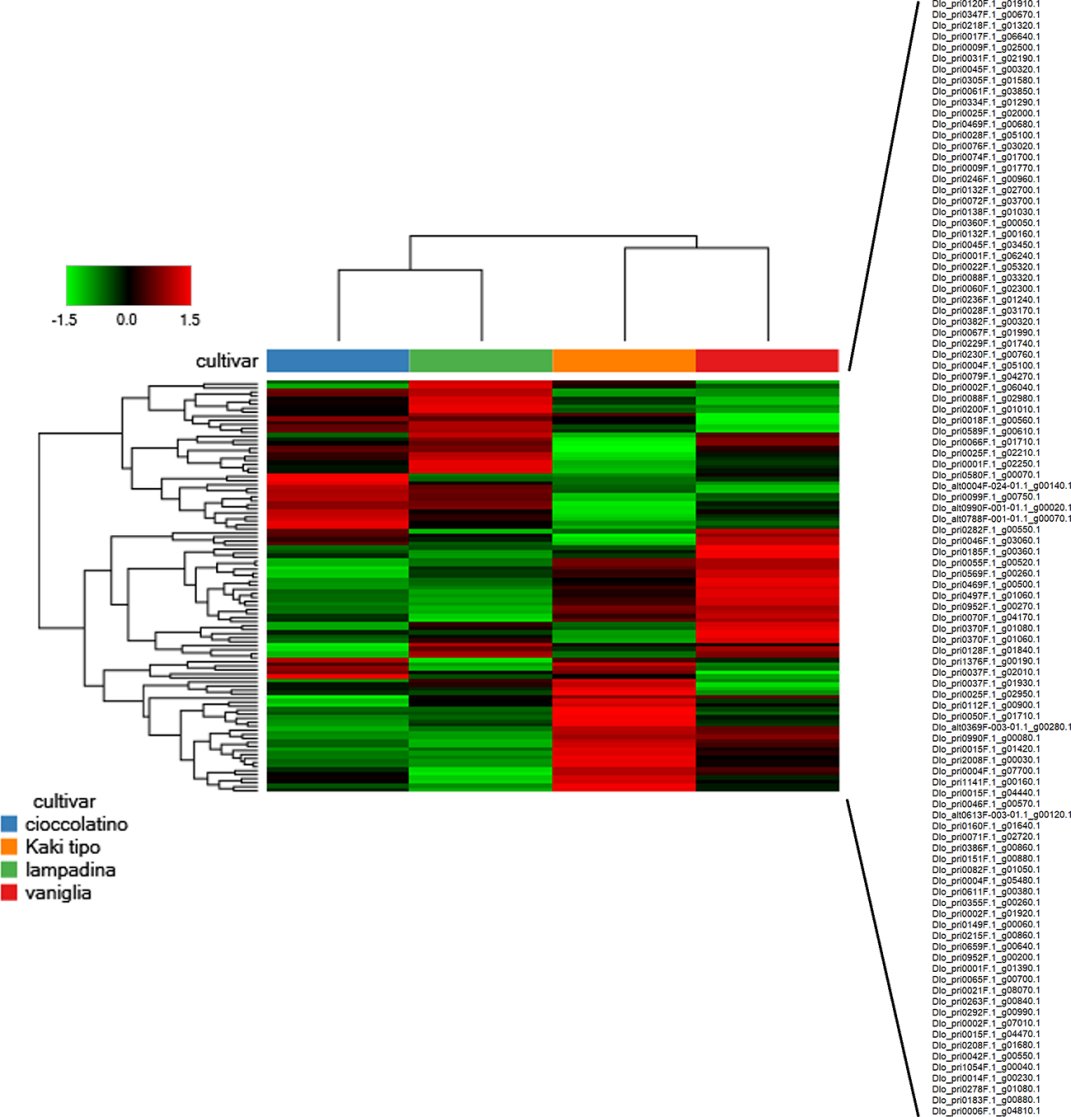
Heat-map showing the variable abundance of differentially represented proteins in persimmon fruits of Kaki tipo, Cioccolatino, Vaniglia and Lampadina cultivars. Each column corresponds to a cultivar, whereas each row represents a single protein. Increasing brightness towards red indicates higher protein responses (measured as summed peak areas) and green indicates lower protein responses. Protein abundance values were grouped for three replicates and scaled before clustering. The dendrograms from the unsupervised hierarchical cluster analysis of the columns and the rows (using the Euclidean distance function and average linkage method) illustrate the similarity of the cultivars and proteins. Protein accession codes reported on the right side of the figure (102 in number) are vertically ordered according to figure appearance. Detailed information on differentially represented proteins reported in this figure are described in **Supplementary Table S3**.

In the whole, we identified 2255 proteins according to the criteria reported in the experimental section; these proteins are reported in **Supplementary Table S2**, which also illustrates corresponding molecular physicochemical parameters. With the aim to describe the possible relationships among identified fruit proteins in Kaki tipo, Lampadina, Vaniglia and Cioccolatino cultivars, we considered quantitative protein differences between ecotypes. Thus, quantitative data on persimmon proteins were filtered according to a fold change value > ± 1.2 in at least one comparison, considering only significant changes of the abundance ratios (*p*-value ≤ 0.01); these parameters were chosen based on precise and accurate quantitation characteristics of the TMT-based proteomic approach, as determined in previous comparative studies on label-free and label-based procedures (Li et al., 2012; Megger et al., 2014). This approach assigned 102 differentially represented fruit proteins (DRPs) in the above-mentioned cultivars (**Supplementary Table S3**). A Euclidean distance heat-map graph was then built up from the corresponding data matrix of average abundances (rows) in the four persimmon cultivars (columns) (**Figure 2**). In a heat-map, rows and columns are regrouped to keep closer those with similar profiles and each row entry in the data matrix is displayed as a color, allowing to view the representation relationships and patterns graphically (Haarman et al., 2015). Accordingly, each persimmon cultivar showed a characteristic representation profile, with black, red and green boxes representing unchanged, over-represented and down-represented proteins, respectively (**Figure 2**). Most heat-maps use an agglomerative hierarchical clustering algorithm to group the data according to the peculiar profiles through a dendrogram. When two clusters are merged, a line is drawn connecting the two clusters at a height corresponding to how similar the clusters are. The heat-map representation of persimmon DRPs showed that Vaniglia and Kaki tipo ecotypes clustered together, similarly to what Cioccolatino and Lampadina independently did (**Figure 2**). The distinctions and similarities clustering the proteomic profiles of the studied persimmon ecotypes were associated with the phenotypic features of each variety, possibly leading to a discrimination of the different accessions.

**Figure 3:**
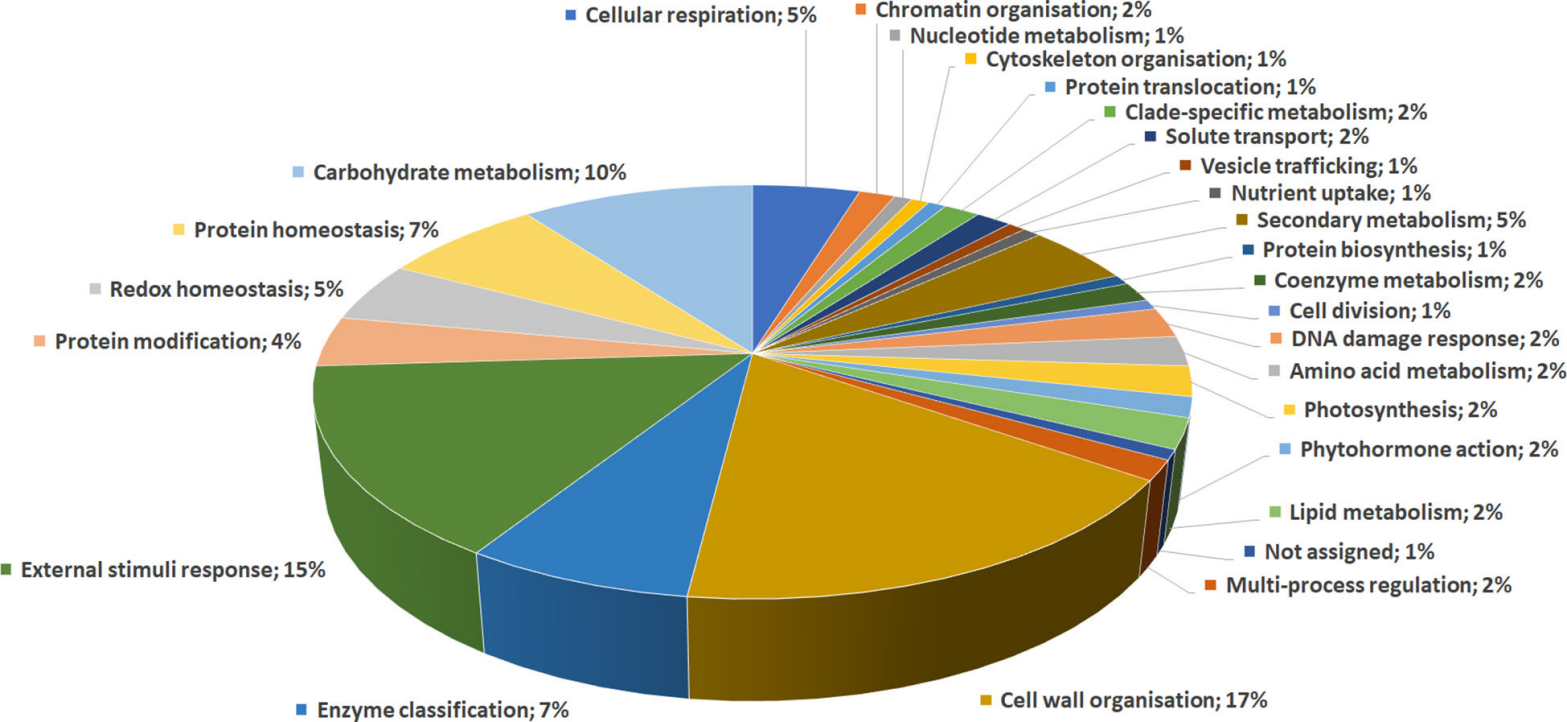
Functional distribution of differentially represented proteins in persimmon fruits of Kaki tipo, Cioccolatino, Vaniglia and Lampadina cultivars. Identified protein species were initially assigned with Mercator software, followed by a functional group cataloguing including information from recent literature data.

DRPs were indexed by an initial functional assignment obtained by Mercator software analysis integrated with information from recent literature (**Supplementary Table S3**). All proteins were associated with one/multiple function(s), except one that was not associated with a known role. According to their identity, these proteins were related to: i) cell wall organization (17%); ii) external stimuli response (15%); iii) carbohydrate metabolism (10%); iv) protein homeostasis (7%); v) enzyme classification (7%); vi) secondary metabolism (5%); vii) redox homeostasis (5%); viii) cellular respiration (5%), underlining the prominent fruit biochemical mechanisms differentiating persimmon ecotypes (**Figure 3**).

Regarding proteins showing specific differences in representation among cultivars, worth mentioning are a number of enzymes involved in cell wall organization, including pectin methylesterase, β-galactosidase, pectate lyase, some xyloglucan endotransglycosylase/hydrolase isoforms, and bifunctional α-L-arabinofuranosidase/β-D-xylosidase isoforms (**Supplementary Figure S1A**), which have already been reported regulating fruit firmness in persimmon and other fruits (Di Santo et al., 2009; Kou et al., 2021a). Their quantitative levels possibly contributed to firmness of fruits, which however altogether did not show significant differences among cultivars (**Table 1**). In this context, variable expression of genes related to cell wall organization was already described differentiating fruits of other non-Italian *D. kaki* cultivars (Kou et al., 2021a). Functionally related to these enzymes are also some proteins involved in carbohydrate metabolism, cell respiration and ultimately to acetaldehyde degradation/ethanol formation, including beta-fructofuranosidase, alpha-galactosidase, galactose mutarotase, beta-glucosidase isoforms, sucrose synthase, dTDP-4-dehydrorhamnose 3,5-epimerase, UDP-D-glucose 6-dehydrogenase, trehalase, fructose kinase isoforms, phosphofructokinase, glyceraldehyde 3-phosphate dehydrogenase and alcohol dehydrogenase (**Supplementary Figure S1B**). Acetaldehyde, ethanol and some of the above-mentioned enzymes have already been reported regulating PAs degradation and astringency in persimmon (Luo et al., 2014; Chen et al., 2017; Kou et al., 2021b; Wu et al., 2022). Their differential representation could possibly account for the slight changes in total sugar content between cultivars (**Table 1**) (Giordani, 2002; Insero et al., 2002b). Deregulated expression of genes related to carbohydrate metabolism, cell respiration and acetaldehyde degradation/ethanol formation was already reported as a discriminant parameter differentiating Chinese and Japanese *D. kaki* cultivars (Nishiyama et al., 2018; Kou et al., 2021b; Zheng et al., 2021).

A similar consideration can also be made for enzymes involved in redox homeostasis, such as some glutathione S-transferase isoforms, thioredoxin, nucleoredoxin and glutathione peroxidase (**Supplementary Figure S2A**), which regulate the concentration of various reducing species, including glutathione, ultimately affecting the antioxidant properties of diverse fruits. Regarding enzymes involved in secondary metabolism, significant was also the differential representation of isoflavone reductase, aureusidin synthase isoforms and UDP-glycosyltransferase superfamily protein involved in flavonoid biosynthesis (Ono et al., 2006; Akagi et al., 2009), as well as z-carotene desaturase catalyzing conversion of z-carotene in lycopene (**Supplementary Figure S2B**). Flavonoids and carotenoids affect the final color of fruits; thus, above-mentioned enzymes may contribute to determine the observed fruit pigment differences between persimmon cultivars (**Table 1** and **Figure 1**). These expression differences of genes related to redox homeostasis and secondary metabolism resemble those already described in fruit of other non-Italian *D. kaki* cultivars (Nishiyama et al., 2018; Zheng et al., 2021).

**Figure 4:**
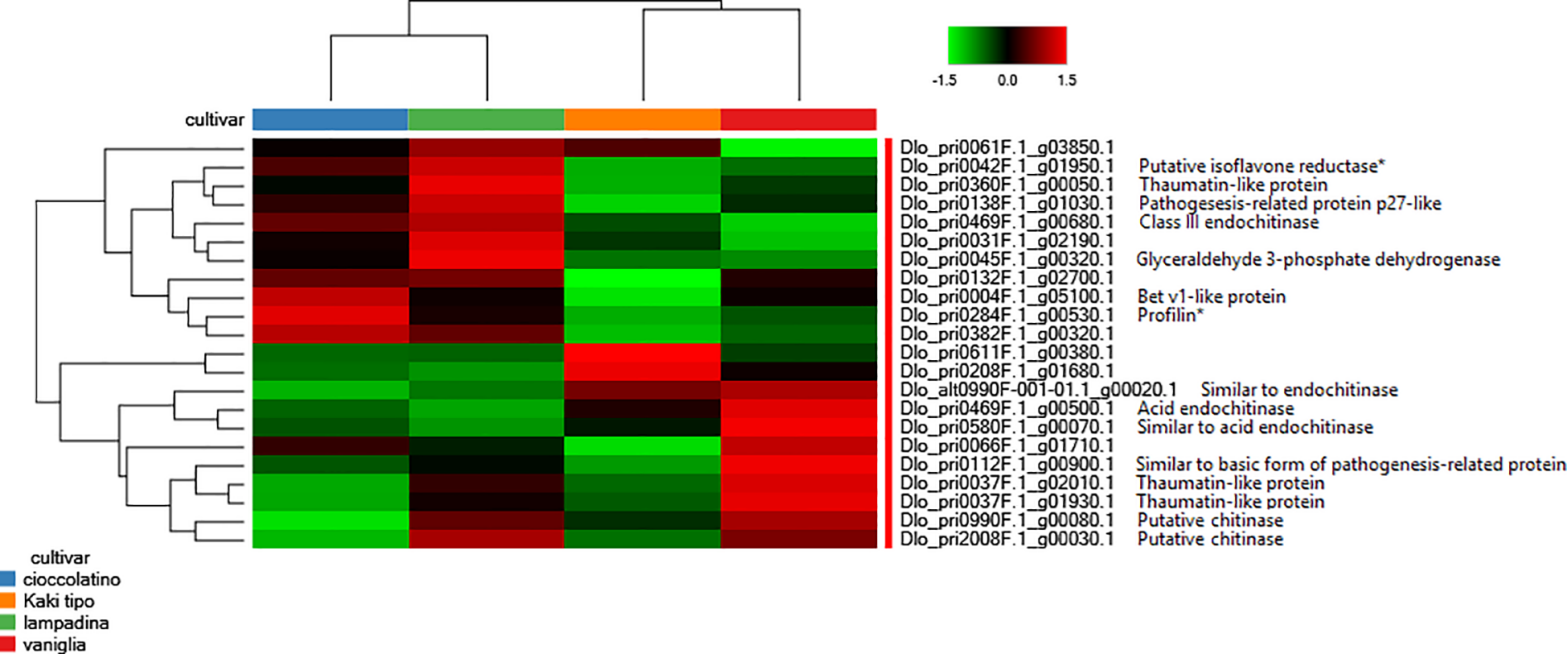
Heat-map reporting the variable abundance of differentially represented proteins involved in response to external stimuli and allergenicity in persimmon fruits of Kaki tipo, Cioccolatino, Vaniglia and Lampadina cultivars. Each column corresponds to a cultivar, whereas each row represents a single protein. Increasing brightness towards red indicates higher protein responses (measured as summed peak areas) and green indicates lower protein responses. Protein abundance values were grouped for three replicates and scaled before clustering. Known allergenic components present in persimmon and other fruits (https://www.allergome.org/) included isoflavone reductase (dlo_pri0042f.1_g01950.1), some thaumatin-like proteins (dlo_pri0360f.1_g00050.1, dlo_pri0037f.1_g02010.1 and dlo_pri0037f.1_g01930.1), some pathogenesis-related proteins (dlo_pri0138f.1_g01030.1 and dlo_pri0112f.1_g00900.1), some endochitinases (dlo_pri0469f.1_g00680.1, dlo_pri0469f.1_g00500.1, dlo_pri0580f.1_g00070.1, and dlo_alt0990f-001-01.1_g00020.1), glyceraldehyde 3-phosphate dehydrogenase (dlo_pri0045f.1_g00320.1), Bet v1-like protein (dlo_pri0004f.1_g05100.1), profilin (dlo_pri0284f.1_g00530) and some chitinases (dlo_pri0990f.1_g00080.1 and dlo_pri2008f.1_g00030.1). Detailed information on the remaining differentially represented proteins reported in this figure are described in **Supplementary Table S3**. Asterisk indicates allergenic proteins whose differential representation was assigned based on the significance of change in at least one comparison (*p*-value ≤ 0.01).

Worth mentioning were also the differential levels measured in various accessions of proteins involved in plant response to external stimuli, including some endochitinase isoforms, thaumatin-like protein isoforms, pathogenesis-related protein p27-like, basic form of pathogenesis-related protein 1 and Bet v1-like protein, the latter being the homologue form of the major allergen Pru ar 1 (**Figure 4**). Genes related to these proteins have been recently demonstrated being differentially expressed when fruits from non-Italian *D. kaki* cultivars were compared (Zheng et al., 2021; Kou et al., 2021b). Along with glyceraldehyde 3-phosphate dehydrogenase, profilin and isoflavone reductase, some thaumatin-like proteins, Bet v1-like protein and endochitinases are well-known allergenic components present in persimmon and other fruits (https://www.allergome.org/) (Karamloo et al., 2001; Bolhaar et al., 2005; Ballmer-Weber and Hoffmann-Sommergruber, 2011; Tsai et al., 2017; Chebib et al., 2022). **Figure 4** summarizes the relative representation of allergenic proteins in fruits of the persimmon accessions here investigated, with possible implications on the allergic impact of corresponding pomes.

**Figure 5:**
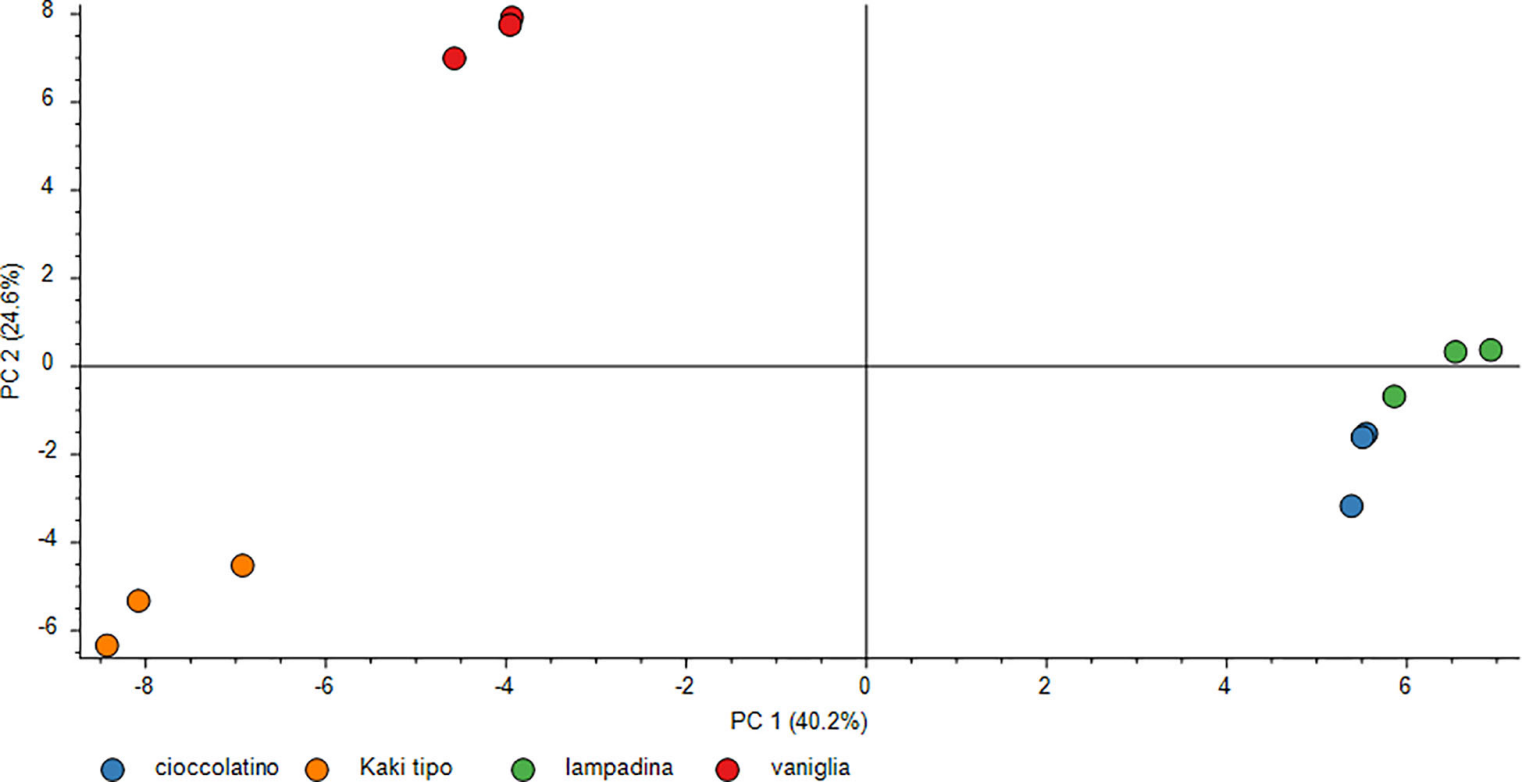
PCA score plot of differentially represented proteins in persimmon fruits of Kaki tipo, Cioccolatino, Vaniglia and Lampadina cultivars. This plot demonstrates the separation of the protein level data (normalized abundances) and the genotype dependent relationship. Each point in the chart describes a biological replicate. PCA explained about 64.8% of variability among persimmon cultivars.

To obtain an overall image of the data interpretation as well as to visualize the above-mentioned proteomic differences among four persimmon cultivars, we also performed PCA analysis of the normalized protein abundances among ecotypes; results are shown in **Figure 5**. PCA of all DRPs evidenced a higher similarity for Lampadina and Cioccolatino persimmon proteomes, which showed evident differences with respect to Vaniglia and Kaki tipo ones, the latter highlighting a significant resemblance between each other. Indeed, loading plot of variables and factors magnified differences among cultivars revealing an effective spatial distribution and local proximity between Lampadina and Cioccolatino cultivars.

### 3.3 Metabolomic analysis

Solid phase extraction coupled to LC-ESI-MS/MS was optimized to further discriminate persimmon cultivars focusing on phytochemicals as polyphenols. The use of the internal standard 4-butyl-hydroxybenzoate improved the protocol of metabolite extraction, leading to an average molecular recovery higher than 90%. Compounds were screened in an *in-house* database including over 250 polyphenols spectra manually annotated from raw files and matched with accessible database listed in the experimental section. Specifically, 33 analytes were identified considering their chromatographic behavior, molecular formula and a mass accuracy lower than ± 5 ppm; results are reported in **Table 2**. The most abundant compounds in the four cultivars were catechin, caffeic acid hexoside and vanillic acid hexoside, confirming previous results on the concentration of flavanol derivatives in persimmon fruits (de Pascual-Teresa et al., 2000). **Supplementary Figure S3** shows the MS/MS spectra of the most representative analytes. Along with the precursor ion [M-H]^-^ at *m/z* 329.08780, the typical fragmentation spectrum of vanillic acid hexoside (**Supplementary Figure S3A**) showed the abundant [M-138]^-^ fragment at *m/z* 191.03, due to the loss of 2-methoxy-4-methylphenol, in accordance with data reported in massbank (www.massbank.eu), as well as [M-18]^-^ and [M-162]^-^ fragments at *m/z* 311.08 and 167.03, due to the loss of water and hexose, respectively. The latter fragment was associated with the aglycone form of vanillic acid, and further yielded [M-44]^-^ fragment at *m/z* 123.04 as result of the loss of CO_2_ from the cyclic structure. Moving to hydroxycinnamic acid derivatives, the MS/MS spectrum of coumaric acid hexoside (**Supplementary Figure S3B**) similarly exhibited the [M-H]^-^ ion at *m/z* 325.09290, and [M-18]^-^ and [M-162]^-^ fragments at *m/z* 307.33 and 163.04 deriving from the loss of water and hexose, respectively; the latter fragment was associated with corresponding aglycone form, and showed the typical fragment due to the further loss of CO_2_ at *m/z* 119.05. As shown in **Supplementary Figure S3C**, the most abundant component of flavanol sub-class, namely catechin, exhibited abundant [M-44]^-^ and [M-84]^-^ fragments at *m/z* 245.08 and 205.05, which were related to the molecular loss of -CH_2_-CHOH group/CO_2_ and the A ring, respectively. The fragmentation spectrum also showed two less intense [M-18]^-^ and [M-110]^-^ fragments at *m/z* 271.06 and 179.03 associated with the loss of water, both in line with the fragmentation patterns previously described (Stoggl et al., 2004). **Supplementary Figure S3D** shows the fragmentation spectrum of phloretin hexoside; the corresponding assigned aglycon form [M-162]^-^ at *m/z* 273.08, the typical fragment of phloretin at *m/z* 167.03 as well as the [M-138]^-^ fragment at *m/z* 297.08 were in agreement with previous observations (Mena et al., 2012).

**Table 2:** Polyphenols identified in negative mode [M-H]^-^ in the fruit of Kaki tipo, Cioccolatino, Vaniglia and Lampadina cultivars.

Compound	Rt	Theoretical mass	Accuracy (ppm)	MS/MS ion fragments (m/z)
Hydroxycinnamic acids				
Caffeic acid	5.7	179.03498	2.96	134 (100%)
Sinapic acid	6.7	223.06120	-2.24	193 (100%), 149 (28%)
Coumaric acid	6.9	163.04007	3.68	119 (100%)
Chlorogenic acid	6.4	353.08781	-2.75	191 (100%)
Dicaffeoylquinic acid	9.8	515.11950	1.75	353 (100%), 191 (8%)
Caffeic acid-hexoside	5.2	341.08780	-3.31	179 (100%), 135 (11%)
Coumaric acid hexoside	6.0	325.09290	3.38	145 (100%), 163 (80%), 187 (40%), 265 (20%), 119 (20%), 205 (10%)
Ferulic acid hexoside	6.5	355.10345	2.82	193 (100%), 217 (44%), 175(18%), 295 (11%), 235 (5%), 265 (3%)
Hydroxybenzoic acids				
Gallic acid	3.9	169.01425	1.18	125 (100%)
Protocatechuic acid	3.4	153.01933	2.74	109 (100%)
Vanillic acid	8.0	167.03498	3.71	123(100%), 149 (33%)
Hydroxybenzoic acid hexoside	2.9	299.07724	1.50	137 (100%)
Protocatechuic acid hexoside	4.0	315.07216	1.27	153 (100%), 109 (34%)
Vanillic acid hexoside	6.8	329.08781	-1.37	167 (100%), 152 (11%)
Flavanols				
Catechin	5.7	289.07176	2.21	245 (100%), 205 (35%)
Epicatechin	6.8	289.07176	2.91	245 (100%), 205 (55%), 179 (21%), 161 (18%)
Procyanidin B1	5.2	577.13515	1.21	425 (100%), 407 (75%), 289 (17%)
Procyanidin B2	6.0	577.13515	1.25	425 (100%), 407 (75%), 289 (17%)
Epigallocatechin	3.7	305.06668	-1.34	179 (100%), 221 (75%), 261 (40%), 125 (40%), 165 (35%), 137 (25%)
Epigallocatechin 3-O-gallate	6.3	457.07763	1.36	303 (31%), 179 (100%)
Catechin 3-O-gallate	8.3	441.08272	0.75	330 (100%), 161 (50%), 397 (50%), 206 (25%)
Prodelphinidin dimer B3	10.5	609.12498	1.48	301 (100%), 463 (50%), 445 (10%)
Flavonols				
Quercetin hexoside	8.1	463.08820	3.89	301 (100%)
Quercetin 3-O-rutinoside	8.2	609.14611	3.43	301 (100%), 271 (10%)
Quercetin 3-O-rhamnoside	8.7	447.09328	3.13	271 (100%), 243 (50%), 255 (38%) 227 (33%)
Kaempferol 3-O-rutinoside	8.6	593.15119	1.38	285 (100%), 561 (23%), 253 (23%)
Kaempferol hexoside	9.5	447.09328	2.77	284 (100%), 285 (80%) 327 (25%), 255 (10%)
Dihydroquercetin hexoside	5.8	465.10385	1.23	285 (100%), 417 (31%), 241 (22%), 303 (2%)
Dihydroquercetin 3-O-rhamnoside-hexoside	6.3	611.16176	-0.92	285 (100%), 485 (33%), 475 (30%), 501 (28%), 241 (23%), 303 (4%)
Dihydrochalcones				
Phloretin hexoside	9.0	435.12967	-1.84	273 (100%), 297 (20%), 167 (5%)
Phloretin	9.5	273.07685	4.39	167 (100%),116 (25%)
Flavanones				
Naringenin hexoside 1	7.9	433.11402	0.53	271 (100%), 151 (27%), 313 (10%)
Naringenin hexoside 2	8.6	433.11402	0.53	271 (100%), 151 (27%), 313 (10%)

Polyphenols were included in different sub-classes according to Phenol-explorer database. Experimental mass values were obtained with an accuracy of ± 5 ppm, with respect to theoretical counterparts. Compounds identification was achieved in data-dependent scanning mode with specific relative intensities.

Each of the 33 compounds mentioned above contributed to the definition of six polyphenol sub-classes, namely hydroxybenzoic acid derivatives, flavanols, hydroxycinnamic acid derivatives, flavonols, flavanones and dihydrochalcones. **Table 3** reports the specific contribution of polyphenol sub-classes to the overall intensities; despite the contribution of catechin, caffeic acid hexoside and vanillic acid hexoside, hydroxybenzoic acids covered the highest percentage in fruits from all cultivars, ranging from 30% to 44%. Flavanols and hydroxycinnamic acids represented the second and third most abundant sub-classes, respectively, with two exceptions: in the case of Kaki tipo cultivar, hydroxycinnamic acids were higher than flavanols, which accounted for the half of intensities toward the other three cultivars; for Vaniglia accession, the percentage of hydroxycinnamic acids was only 6%, significantly lower than that of the remaining cultivars. The role of hydroxycinnamic acids and flavanols is of primary importance when dealing with fruit acceptability and quality; both polyphenol classes have already been related to fruit astringency according to their degree of polymerization and interaction with mucosal proteins (Bajec and Pickering, 2008).

**Table 3:** Percentage contribution of each compound sub-class to whole polyphenolics present in the fruit of Kaki tipo, Cioccolatino, Vaniglia and Lampadina cultivars.

Sub-classes	Hydroxybenzoic acids	Flavanols	Hydroxycinnamic acids	Flavonols	Flavanones	Dihydrochalcones
**Cioccolatino**	35%^b^	27%^b^	19%^b^	8%^a^	4%^a^	8%^c^
**Vaniglia**	40%^a^	33%^a^	6%^c^	6%^b^	2%^b^	13%^a^
**Lampadina**	30%^c^	28%^b^	23%^a^	7%^b^	4%^a^	7%^c^
**Kaki tipo**	44%^a^	15%^c^	20%^b^	7%^b^	3%^a^	11%^b^

The ratio was calculated by considering the area counts in FTMS mode of the current ion associated with each precursor species listed in **Table 2**. Differences between polyphenol classes within the four persimmon cultivars were analyzed by ANOVA with a confidence interval of 95%; mean values were compared by Tukey’s test (α = 0.05) using XLStat statistical software (Addinsoft, New York, NY, USA). Different letters indicate a significant difference within the same polyphenol class (Tukey’s test, P < 0.05).

**Figure 6:**
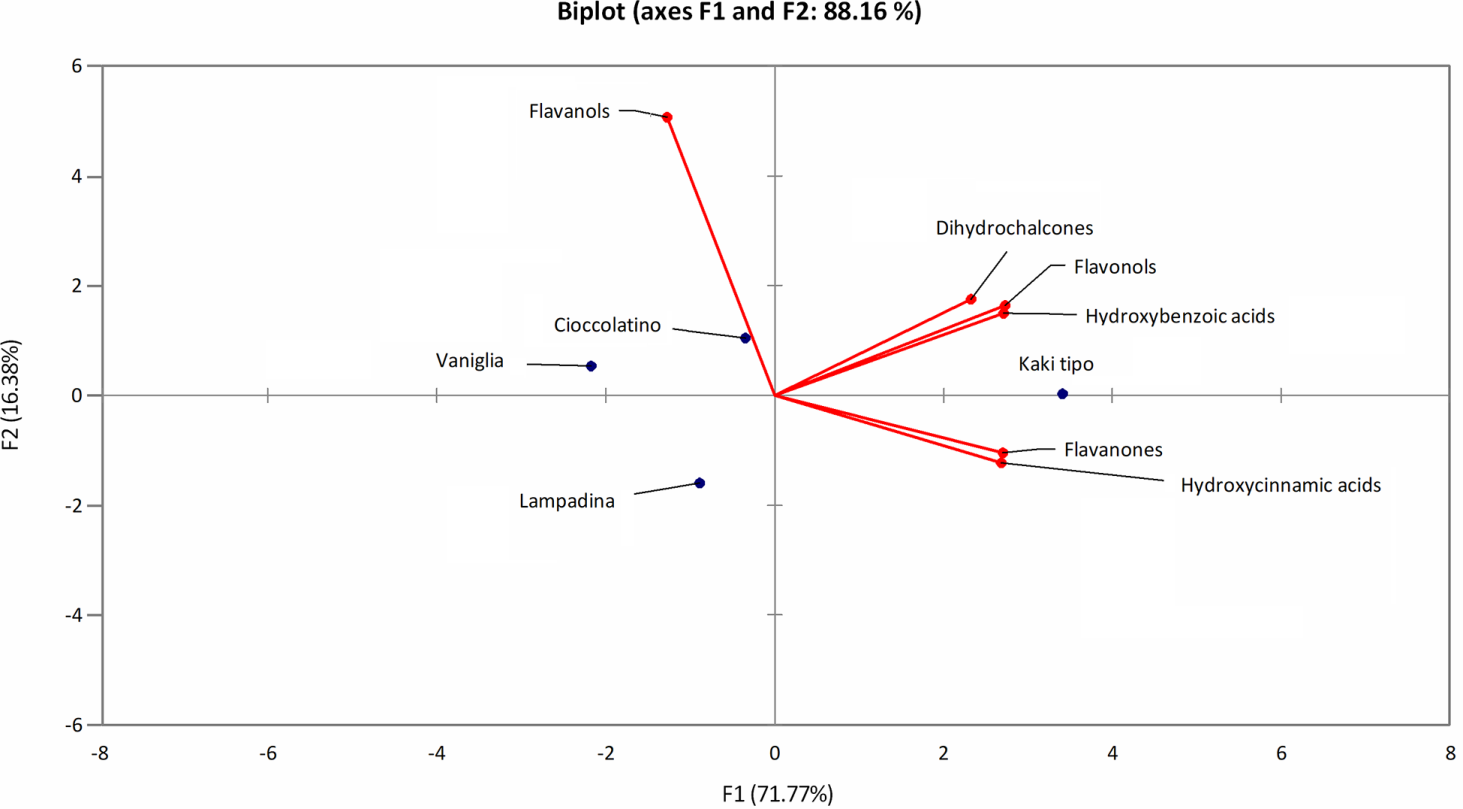
PCA score plot of polyphenols in persimmon fruits of Kaki tipo, Cioccolatino, Vaniglia and Lampadina cultivars. PCA explained about 88.2% of variability among the four cultivars, considering the first two components on x and y axes. Spatial 2D distribution of samples was achieved by considering loading plots of secondary metabolites sub-classes.

The polyphenol contribution to each cultivar is clearly outlined in **Figure 6**. PCA describes how polyphenol sub-classes contributed to the spatial distribution in 2D of each cultivar. While the input of dihydrochalcones, flavonols and hydroxybenzoic acids represented the characteristic trait of Kaki tipo cultivar, flavanols mostly contributed to the molecular characterization of the remaining three accessions, pointing out to polyphenols defining the molecular signature of each ecotype. These results confirmed previous observations describing a differential representation of polyphenols in the fruit of various persimmon cultivars because of genetic traits (Ancillotti et al., 2018; Maulidiani et al., 2018; Esteban-Munoz et al., 2020).

**Figure 7:**
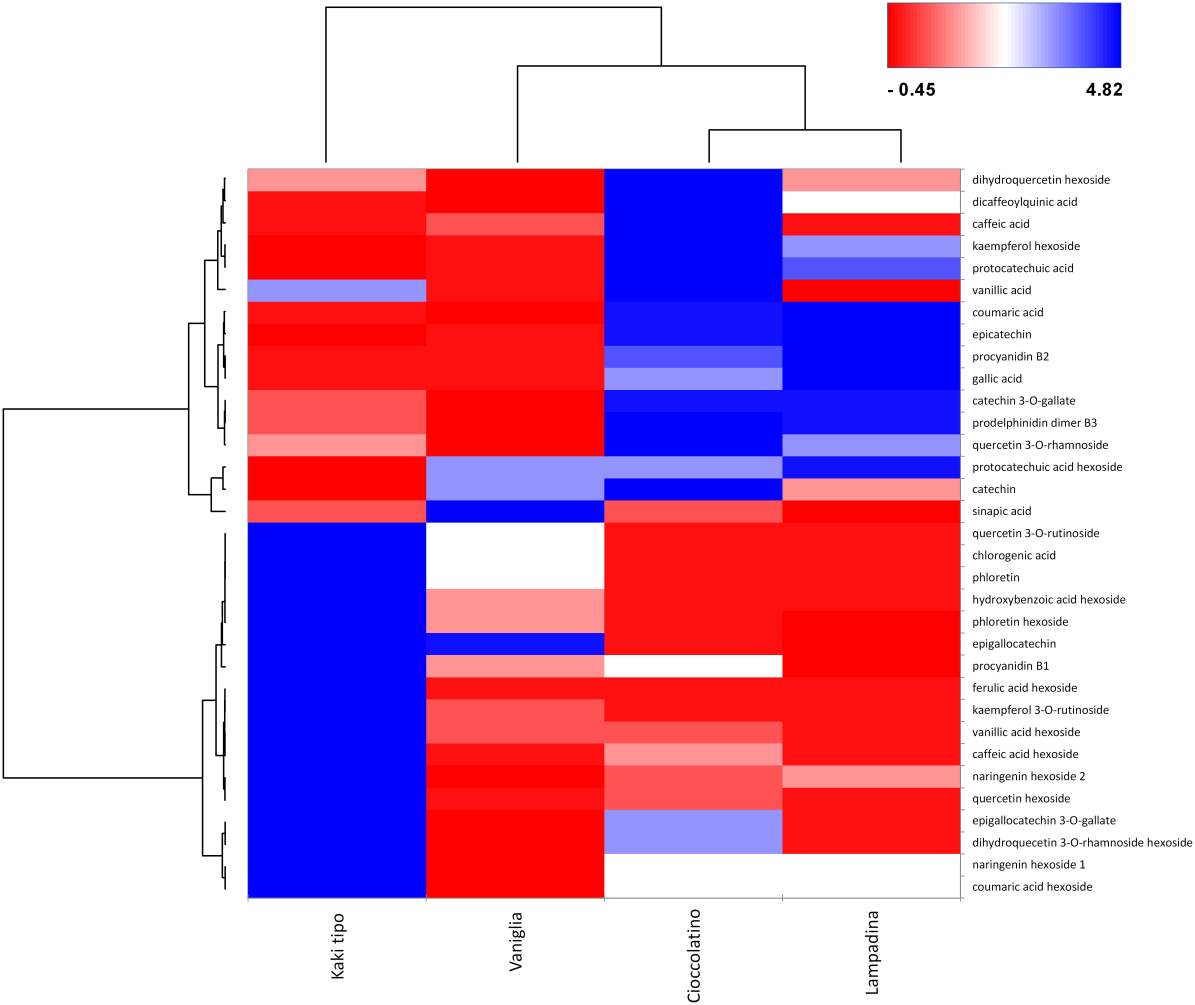
Heat-map reporting the variable abundance of polyphenols in persimmon fruits of Kaki tipo, Cioccolatino, Vaniglia and Lampadina cultivars. Each column corresponds to a cultivar, whereas each row represents a compound. Heat-map encompassed centered and reduced intensities (area counts) moving from red to blue, through white, including Euclidean distance function and Ward linkage method. The dendrograms from the hierarchical cluster analysis of the columns and the rows illustrate the similarity of the cultivars and the distribution of polyphenolic compounds between aglycones and glycosides (top and bottom section), respectively.

In relationship with PCA, heat-map including Euclidean distance function and Ward linkage method depicted tailored discrimination among biomarkers and cultivars (**Figure 7**). Looking at the dendrogram on the x axis, three cultivars were clearly separated with respect to Kaki tipo accession by the first nodal point, which in turn grouped Vaniglia, Cioccolatino and Lampadina ecotypes. Moving forward, a second nodal point separated Vaniglia accession from the cultivars Cioccolatino and Lampadina, which finally clustered together, thus highlighting key similarities between Kaki tipo and Vaniglia ecotypes. Cultivar clustering from metabolomic data well overlapped that from proteomic results (**Figure 2**), clearly distinguishing the association of Cioccolatino and Lampadina ecotypes, from the one of Vaniglia and Kaki tipo accessions.

Polyphenol molecular patterns were described by the dendrogram grouping on y axis. Two groups were separated according to centered and scaled intensities (**Figure 7**): a red intense region in the right down corner grouped the 3 cultivars with lower intensities of hexosides, namely quercetin hexoside, ferulic acid hexoside, caffeic acid hexoside, kampferol rutinoside and vanillic acid hexoside. Such compounds were over-represented in Kaki tipo cultivar. Conversely, the blue region in the right top corner grouped the Cioccolatino and Lampadina cultivars (**Figure 7**), highlighting similar intensities for epicatechin, coumaric acid, catechin gallate, prodelphinidin and procyanidin B2. The peculiar distribution of polyphenols in persimmon cultivars was in line with the differential protein representation of isoflavone reductase therein. The latter enzyme catalyzes the biosynthesis of proanthocyanidin through an integrated metabolic loop affording precursor monomers catechin and epicatechin (Tanner et al., 2003). Over-represented levels of isoflavone reductase in Cioccolatino and Lampadina cultivars perfectly matched to the highest concentrations of above-mentioned metabolites in above-mentioned accessions.

## 4 Conclusions

Proteomics and metabolomics allow large-scale analysis of molecules from biological tissues/fluids, generating holistic information for the characterization of an organism at a specific time of its life. Accordingly, comparative gel-based or label-free proteomic and metabolomic studies were separately used in the past to detail molecular peculiarities of plant cultivars grown under identical experimental conditions, providing punctual information on their biodiversity. These holistic approaches have found interesting applications for plants whose genome was not sequenced, in which intraspecies genetic comparisons generally are limited to comparative analysis of selected genes and/or nucleotide fragments.

In this study, we focused our attention on persimmon and, particularly, on four Italian cultivars whose fruits are largely diffused for food consumption. We originally verified that TMT-based quantitative proteomic procedures can be successfully used to evaluate phenotypic differences between plant ecotypes, estimate corresponding genetic variability, and establish their genetic distance. Our integrated quantitative proteomic and metabolomic results showed peculiar molecular profiles for each cultivar, with Cioccolatino and Lampadina ecotypes showing specific phenotypic differences with respect to Kaki typo and Vaniglia accessions. Similarities among cultivars were also observed through clustering of corresponding data. Similar phenotypic analogies between ecotypes were observed whenever proteomic or metabolomic data were independently used, allowing an autonomous verification of results obtained with a single approach. Accordingly, this integrated procedure can represent a novel key for typing persimmon biodiversity, in which a specific quantitative set of data on proteins/metabolites can be distinctive of a certain cultivar. Even interesting, the above-mentioned goal however requires the screening of a wider set of plant varieties to understand the proteome and metabolome diversity across the *D. kaki* genus. In conclusion, this study paves the way to a novel approach for the characterization of persimmon fruits, moving the attention from usual pomological and genetic parameters to molecular ones, i.e. proteins and metabolites.

## Data availability statement

The datasets presented in this study can be found in online repositories. The names of the repository/repositories and accession number(s) can be found in the article/**Supplementary Material**.

## Author contributions

AMS and AS designed the research. MP, AN, DC and AM sampled fruits of cultivar accessions, performed phenological/agronomic description of plants and characterized physicochemical parameters of corresponding fruits. SP and AMS performed proteomic analysis. SP, AMS and AS performed the bioinformatic and statistical analyses of proteomic data. SP and AT performed metabolomic analysis. SP, and AT performed the bioinformatic and statistical analyses of metabolomic data. SP, AT, MP, AMS and AS drafted the paper. SP, AT, MP, AMS and AS helped to revise the manuscript, with all authors contributing to the discussion of the data. AS provided funds for the research. All authors contributed to the article and approved the submitted version.
