# Supplementary material for: Investigating phenotypic relationships in persimmon accessions through integrated proteomic and metabolomic analysis of corresponding fruits
Source: Front Plant Sci. 2023 Jan 30;14:1093074. doi: 10.3389/fpls.2023.1093074 (PMC9923171; doi:10.3389/fpls.2023.1093074)
Supplement: Supplementary file 1 [file DataSheet_1.pdf]

INVESTIGATING PHENOTYPIC RELATIONSHIPS IN PERSIMMON ACCESSIONS THROUGH INTEGRATED PROTEOMIC AND METABOLOMIC ANALYSIS OF CORRESPONDING FRUITS

Sabrina De Pascale, Antonio Dario Troise, Milena Petriccione, Angelina Nunziata, Danilo Cice, Anna Magri, Anna Maria Salzano and Andrea Scaloni

SUPPLEMENTARY MATERIAL

Supplementary Information

Legend to Supplementary Tables

Supplementary Table S1. Phenological and agronomic traits of Kaki tipo, Lampadina, Vaniglia and Cioccolatino plant ecotypes as characterized according to International Union for the Protection of New Varieties of Plants (UPOV) descriptors.

Supplementary Table S2. Proteins identified in persimmon fruits of Kaki tipo, Lampadina, Vaniglia and Cioccolatino cultivars. Mass spectrometric data were searched against a non-redundant protein sequence FASTA file available for *D. lotus* deriving from the corresponding genome (Akagi et al., 2020), according to parameters reported in the experimental section. Proteins were considered confidently identified based on at least two sequenced peptides and an individual Mascot Score greater or equal to 30. Results were filtered to 1% false discovery rate. In addition to identification and quantitative parameters, this table also reports protein molecular mass, pI and sequence coverage values.

Supplementary Table S3. Differentially represented proteins identified in persimmon fruits of Kaki tipo, Lampadina, Vaniglia and Cioccolatino cultivars. Proteins identified as reported in Supplementary Table S2 were filtered for corresponding quantitative data based on a fold change value $\geq \pm 1.2$ and p-value ≤ 0.01. This assigned 102 differentially represented proteins (DRPs) in persimmon fruit of various persimmon cultivars. Corresponding quantitative data based on TMT reporter ions are reported in this table according to a color format. Based on results on sequence identity percentages, DRPs were indexed by an initial functional assignment obtained through analysis with Mercator software, whose results are also reported in this table, which were further integrated with information from KEGG analysis of *Arabidopsis thaliana* protein homologues and recent literature. Assignment of *A. thaliana* protein homologues was also based on evaluation of sequence identity. Protein homologues assigned based on analysis with Mercator software and searching the KEGG database are described.

Legend to Supplementary Figures

Supplementary Figure S1. Heat-map showing the comparison of the abundance of the differentially represented proteins involved in cell wall organization (A) and carbohydrate metabolism, respiration, and ethanol production (B) identified in persimmon fruits of Kaki tipo, Cioccolatino, Vaniglia and Lampadina cultivars. Each column corresponds to a cultivar, whereas each row represents a single protein. Increasing brightness towards red indicates higher protein responses (measured as summed peak areas) and green indicates lower protein responses. In panel A, pectin methylesterase (dlo_pri0015f.1_g04440.1), β-galactosidase (dlo_pri0025f.1_g02950.1), pectate lyase (dlo_pri0050f.1_g01710.1), xyloglucan endotransglycosylase/hydrolase isoforms (dlo_pri0001f.1_g01390.1, dlo_pri0009f.1_g01770.1, dlo_pri0215f.1_g00860.1 and dlo_pri0263f.1_g00840.1), cell wall peroxidase isoforms (dlo_pri0031f.1_g02190.1, dlo_pri0382f.1_g00320.1 and dlo_pri0611f.1_g00380.1), arabinogalactan protein (dlo_pri0072f.1_g03700.1), pectin acetylesterase isoforms (dlo_pri0370f.1_g01080.1 and dlo_pri0370f.1_g01060.1), and bifunctional α-L-arabinofuranosidase/β-D-xylosidase isoforms (dlo_pri0001f.1_g02250.1, dlo_pri0004f.1_g05480.1 and dlo_pri0292f.1_g00990.1) are reported. Information on beta-fructofuranosidase (dlo_pri0046f.1_g03060.1), beta-glucosidase (dlo_pri0282f.1_g00550.1), endo-beta-1,4-mannase (dlo_pri0025f.1_g02210.1) and dTDP-4-dehydrorhamnose 3,5-epimerase (dlo_pri0589f.1_g00610.1) are also described. In panel B, beta-fructofuranosidase (dlo_pri0046f.1_g03060.1), alpha-galactosidase (dlo_pri0076f.1_g03020.1), galactose mutarotase (dlo_pri0149f.1_g00060.1), beta-glucosidase isoforms (dlo_pri0282f.1_g00550.1 and dlo_pri0066f.1_g01710.1), sucrose synthase (dlo_pri0015f.1_g04470.1), dTDP-4-dehydrorhamnose 3,5-epimerase (dlo_pri0589f.1_g00610.1), UDP-D-glucose 6-dehydrogenase (dlo_pri0001f.1_g06240.1), trehalase (dlo_pri0185f.1_g00360.1), fructose kinase isoforms (dlo_alt0369f-003-01.1_g00280.1 and dlo_pri0246f.1_g00960.1), phosphofructokinase (dlo_pri0046f.1_g00570.1), glyceraldehyde 3-phosphate dehydrogenase (dlo_pri0045f.1_g00320.1) and alcohol dehydrogenase (dlo_pri0014f.1_g00230.1) are reported. Information on dihydrolipoyl dehydrogenase component L-protein of glycine cleavage system (dlo_pri0006f.1_g04810.1), beta-galactosidase (dlo_pri0021f.1_g08070.1), Rieske Fe-S component of cytochrome c reductase complex (dlo_pri0009f.1_g02500.1) and component NDUFA6/B14 of NADH dehydrogenase alpha subcomplex (dlo_pri0022f.1_g05320.1) are also described.

Supplementary Figure S2. Heat-map showing the comparison of the abundance of the differentially represented proteins involved in redox homeostasis (A) and secondary metabolism (B) identified in persimmon fruits of Kaki tipo, Cioccolatino, Vaniglia and Lampadina cultivars. Each column corresponds to a cultivar, whereas each row represents a single protein. Increasing brightness towards red indicates higher

protein responses (measured as summed peak areas) and green indicates lower protein responses. In panel A, glutathione S-transferase isoforms (dlo_pri0061f.1_g03850.1, dlo_pri0132f.1_g02700.1 and dlo_pri0230f.1_g00760.1), thioredoxin (dlo_pri0183f.1_g00880.1), nucleoredoxin (dlo_pri0079f.1_g04270.1) and glutathione peroxidase (dlo_alt0613f-003-01.1_g00120.1) are reported. In panel B, aureusidin synthase isoforms (dlo_pri0952f.1_g00200.1 and _pri0952f.1_g00270.1), UDP-glycosyltransferase superfamily protein (Dlo_pri0088F.1_g02980.1) and z-carotene desaturase (dlo_pri0347f.1_g00670.1) are reported. Information on tropinone reductase (dlo_pri0128f.1_g01840.1) and salicylic acid-binding protein (dlo_pri0082f.1_g01050.1) are also described.

Supplementary Figure S3. Tandem MS spectra of the four most representative polyphenols detected in persimmon fruits of Kaki tipo, Cioccolatino, Vaniglia and Lampadina cultivars. Spectra were acquired in FTMS mode, with a collision induced dissociation value set at 25. Vanillic acid hexoside, coumaric acid hexoside, catechin and phloretin hexoside are reported in panel A, B, C and D, respectively. Range of *m/z* is specific for each filter according to the retention time and the data dependent scan.

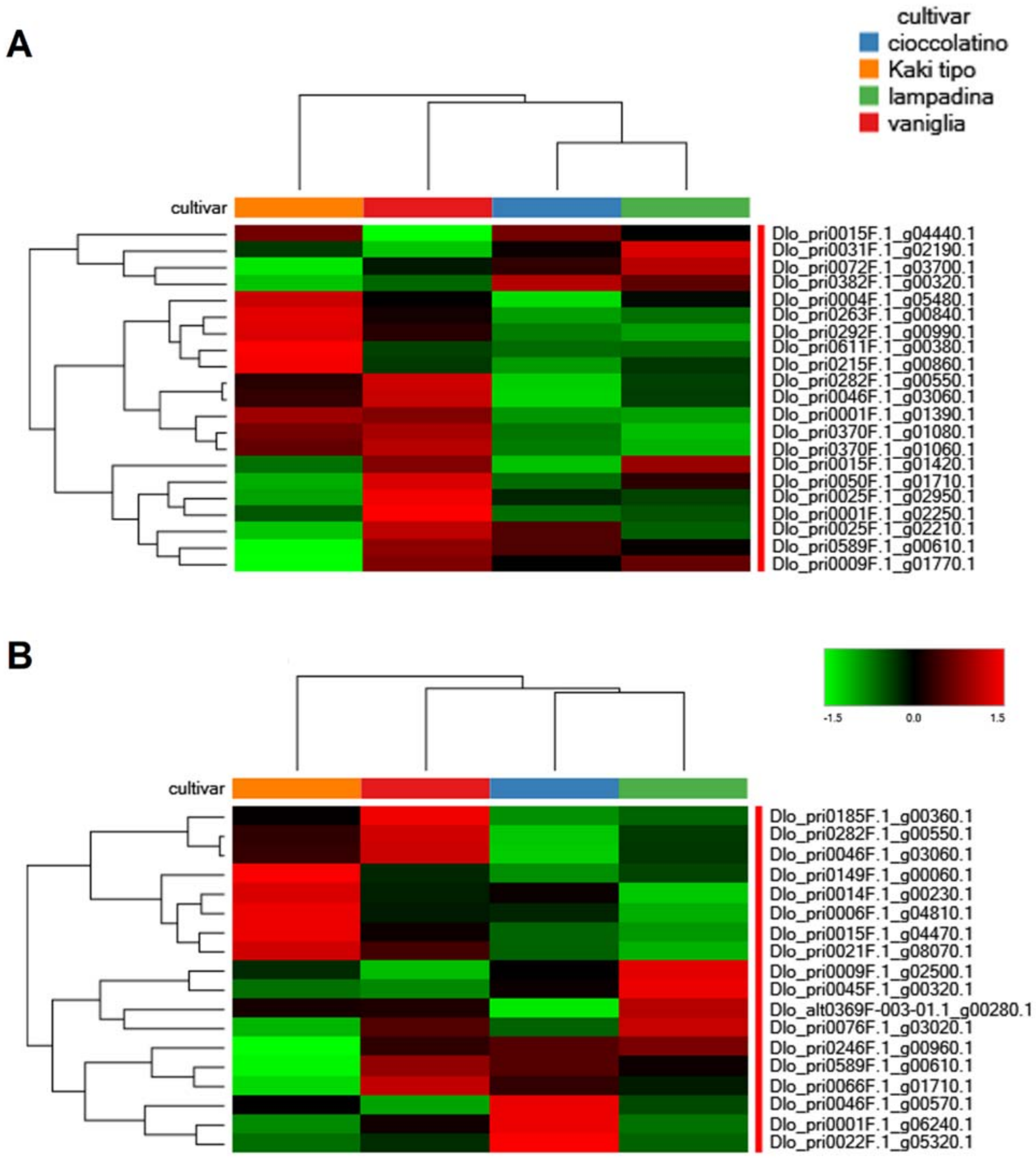

Supplementary Figure S1.

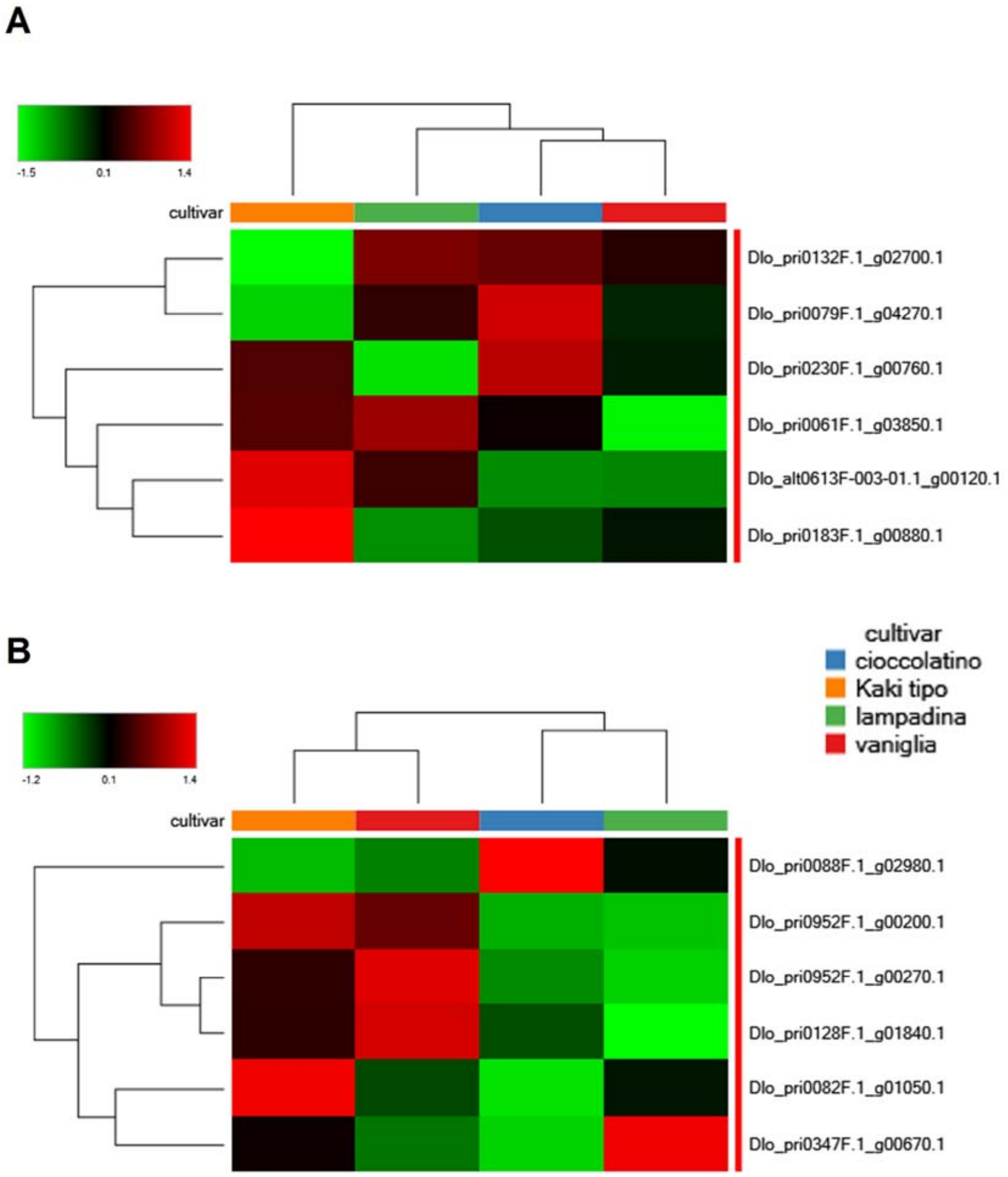

Supplementary Figure S2.

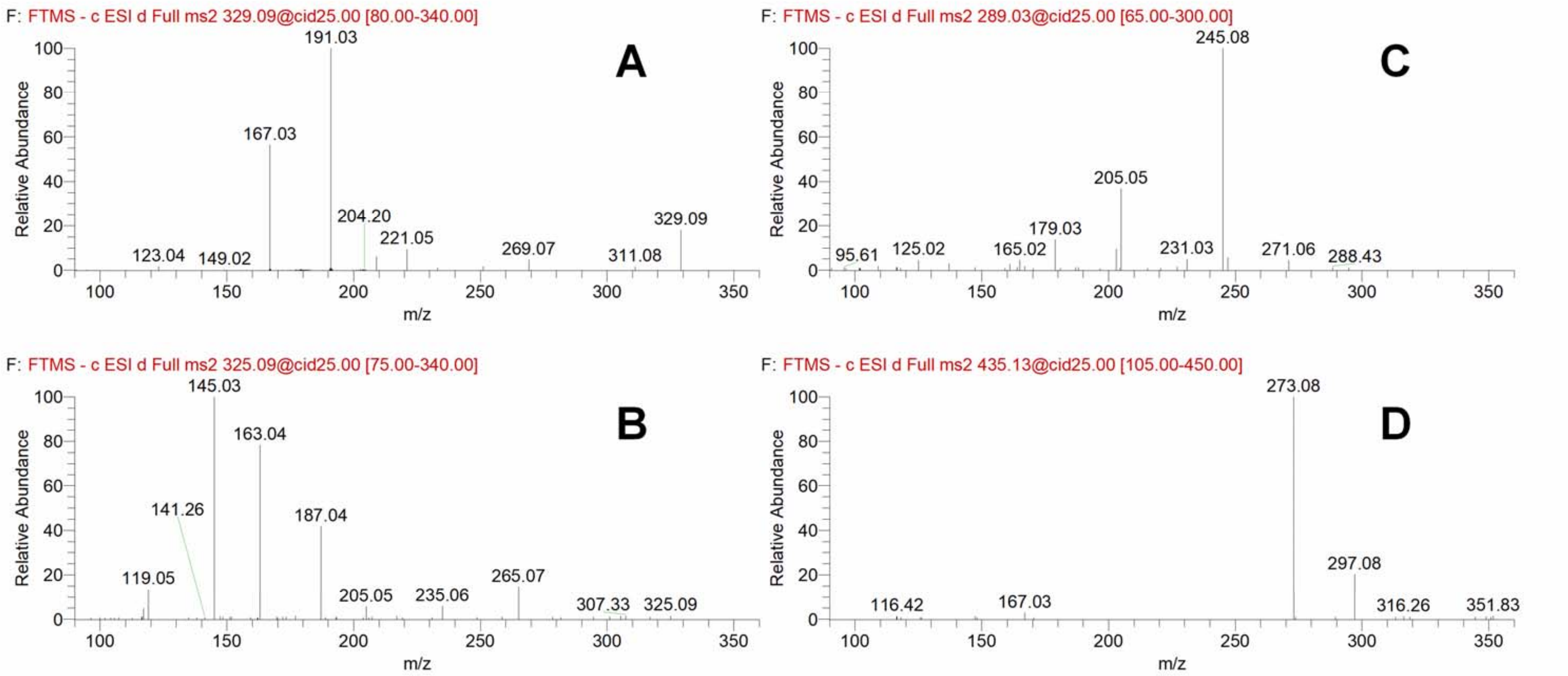

Supplementary Figure S3.
